# Genetic diversity and antimicrobial resistance in *Staphylococcus aureus* and coagulase‐negative *Staphylococcus* isolates from bovine mastitis in Minas Gerais, Brazil

**DOI:** 10.1002/mbo3.736

**Published:** 2018-10-08

**Authors:** Elaine M. S. Dorneles, Mariana D. A. M. Fonseca, Juliana A. P. Abreu, Andrey P. Lage, Maria A. V. P. Brito, Carine R. Pereira, Humberto M. Brandão, Alessandro S. Guimarães, Marcos B. Heinemann

**Affiliations:** ^1^ Departamento de Medicina Veterinária Universidade Federal de Lavras Lavras Minas Gerais Brazil; ^2^ Departamento de Medicina Veterinária Preventiva e Saúde Animal Faculdade de Medicina Veterinária e Zootecnia Universidade de São Paulo São Paulo São Paulo Brazil; ^3^ Departamento de Medicina Veterinária Preventiva Escola de Veterinária Universidade Federal de Minas Gerais Belo Horizonte Minas Gerais Brazil; ^4^ Núcleo de Saúde Animal e Qualidade do Leite Embrapa Gado de Leite Juiz de Fora Minas Gerais Brazil

**Keywords:** antimicrobial resistance, coagulase‐negative staphylococci, methicillin‐resistant *S. aureus*, PFGE, *Staphylococci*

## Abstract

The aims of this study were to determine the antimicrobial susceptibility profile and genetic diversity of *Staphylococcus* spp. isolated from dairy cows in Minas Gerais, Brazil, and to assess the relationship among the isolates’ susceptibility profiles and pulsed‐field gel electrophoresis (PFGE) genotypes. Seventy‐nine isolates were used, including *S. aureus* (*n* = 71) and coagulase‐negative staphylococci (CoNS) (*n* = 8). Susceptibility to 12 antimicrobial agents was performed. All *Staphylococcus* spp. were subjected to PFGE. *Staphylococcus aureus* and CoNS isolates exhibited full susceptibility only to cephalothin. The greatest percentages of resistance among *Staphylococcus* spp. were observed to penicillins, folate pathway inhibitors, and tetracyclines. Twelve *S. aureus* and four CoNS were classified as multidrug resistance strains. Percentage of MRSA was also higher among CoNS (75%), compared to *S. aureus* isolates (2.81%). Adopting 100% of similarity, 34 different genotypes were identified. Association of minimum‐spanning tree (MST) analysis with data from municipalities, herds, methicillin‐resistant *S. aureus* (MRSA), and resistance patterns for all isolates did not show any clustering. However, a clustering pattern of bacterial species was observed. Results from this study indicate a high frequency of antimicrobial resistance, especially among CoNS, and a high genetic diversity among *Staphylococcus* spp. isolated from dairy cows with mastitis in Minas Gerais, Brazil.

## 1 INTRODUCTION

Mastitis is considered the main disease affecting dairy cattle herds in the world, being responsible for major economic losses to the dairy sector (Ruegg, 2012). In Brazil, as well as in several other countries, *Staphylococcus aureus* and *Streptococcus agalactiae* are considered the most common agents causing mastitis in cattle (Acosta, Silva, Medeiros, Pinheiro‐Júnior, & Mota, 2016). These pathogens can colonize the mammary gland of ruminants, where they can survive for long periods causing mild or no clinical symptoms. From the epidemiological point of view, it is critical to define the microorganisms involved in the etiology of the bovine mastitis, to determine the reservoirs and the sources of infection, and to adopt appropriate hygienic measures and a strict monitoring program.

*Staphylococcus aureus* and coagulase‐negative staphylococci (CoNS) are the major agents of contagious bovine mastitis in several countries and are recognized worldwide as a major public health issue, since they cause serious diseases in humans and animals and have great ability to become resistant to antibiotics (Aires‐de‐Sousa, 2017; Botrel et al., 2010; Santos et al., 2016). Methicillin‐resistant *S. aureus* (MRSA), which is resistant to all beta‐lactams antibiotics, are of particular concern, as recently, these strains have also emerged as a widespread cause of infections in the community and in animals, besides hospitals (Chambers & DeLeo, 2009). Moreover, albeit majority of bovine mastitis pathogens are susceptible to the antimicrobials used for treatment, some studies have indicated an increasing resistance among *S. aureus* and especially CoNS to β‐lactam antibiotics, including previous study carried out in *Staphylococcus* strains from Minas Gerais, Brazil (Botrel et al., 2010; Kalmus, Aasmae, Karssin, Orro, & Kask, 2011; Santos et al., 2016).Surveillance of resistant staphylococci and the accurate diagnosis of these bacteria are critical for appropriate mastitis treatment and for the identification of risks to the general population, as strains generated in the livestock environment can spread to the community by contact with infected animals or contaminated animal products.

However, besides the identification of the pathogens involved in mastitis, it is also important to use highly discriminatory methods for the characterization of these isolates, which will allow to trace back the outbreaks, to understand the transmission routes, and to control the disease spread (Boffetta, 2000). The pulsed‐field gel electrophoresis (PFGE) of bacterial isolates from mastitis has shown a high discriminating power, especially for *S. aureus*, being considered the standard method for epidemiological studies of this species (Goering, 2010). This technique has been extensively used for the comprehension of the epidemiology of both endemic and epidemic MRSA strains from different origin (livestock, community, and hospital), contributing for the elucidation of the epidemiology of staphylococcal infection in all regions of the world. Thus, the aims of present study were (a) to determine the antimicrobial susceptibility profile and (b) genetic diversity, using PFGE, of *Staphylococcus* spp. strains isolated from dairy cows with mastitis in Minas Gerais, Brazil, and (c) to assess the relationship among the susceptibility profiles of the isolates, epidemiological data, and PFGE genotypes.

## 2 MATERIALS AND METHODS

### 2.1 Bacterial isolates and culture conditions

Seventy‐nine isolates of *Staphylococcus* spp. (*S. aureus n* = 71 and CoNS *n* = 8) previously isolated from milk samples of dairy cows with mastitis and *S. aureus* ATCC 25923^T^ were used in the present study (Santos et al., 2016).

### 2.2 Antimicrobial susceptibility testing

Susceptibility to 12 antimicrobial agents was performed using the disk‐diffusion method in accordance with the Clinical and Laboratory Standards Institute recommendations VET01‐A4 manual (CLSI 2013b). Antimicrobial agents tested were ampicillin (10 μg), cephalothin (30 μg), ceftiofur (30 μg), clindamycin (2 μg), erythromycin (15 μg), enrofloxacin (5 μg), gentamycin (10 μg), oxacillin (1 μg), penicillin G (10 U), sulfonamide (300 μg), tetracycline (30 μg), and trimethoprim/sulfamethoxazole (1.25/23.75 μg) (all purchased from Oxoid, UK). All antimicrobials were tested with the reference strain *S. aureus* ATCC 25923^T^ to ensure that the results were within acceptable limits of quality control for susceptibility testing according to CLSI document VET01‐A4. Isolates were classified as resistant, intermediate, or sensitive to antimicrobials according to CLSI manual VET01‐S2 (CLSI 2013a). Multidrug resistance was defined as resistance to three or more antimicrobial groups (Magiorakos et al., 2012; Schwarz et al., 2010). The antimicrobial groups were as follows: penicillins (ampicillin, oxacillin and penicillin G); chephems (cephalothin and ceftiofur); lincosamides (clindamycin); macrolides (erythromycin); quinolones (enrofloxacin); aminoglycosides (gentamicin); folate pathway inhibitors (sulfonamide and trimethoprim/sulfamethoxazole); and tetracyclines (tetracycline). Oxacillin resistant *S. aureus* strains were classified as MRSA.

### 2.3 DNA extraction and pulse field gel electrophoresis (PFGE)

Preparation of DNA of *S. aureus* and CoNS strains was performed as described by Mulvey et al. (2001). All *Staphylococcus* spp. isolates were subjected to restriction‐endonuclease digestion and PFGE strictly following the procedures as described by Mork, Tollersrud, Kvitle, Jorgensen, and Waage (2005).

The number and size of the DNA fragments obtained in PFGE were assessed using the software BioNumerics 7.5 (Applied Maths, Belgium). Clustering analysis was performed based on the Dice coefficient and the unweighted pair group method with arithmetic mean (UPGMA) using the same software. The Hunter and Gaston Diversity Index (HGDI) was calculated (Hunter & Gaston, 1988) (http://insilico.ehu.eus/mini_tools/discriminatory_power/index.php). The minimum‐spanning tree (MST) was generated to assess the association of clustering patterns of the isolates and antimicrobial susceptibility profiles, herds, MRSA, or municipalities. MST presented is the one with the highest overall reliability score and was performed using the UPGMA to calculate the distance matrix Prim's algorithm associated with the priority rule and the permutation resampling (Feil, Li, Aanensen, Hanage, & Spratt, 2004).

## 3 RESULTS AND DISCUSSION

*Staphylococcus* spp., particularly *S. aureus*, stands out as one of the most common agents involved in the etiology of bovine mastitis. In addition to veterinary importance, there are also implications in public health due to the potential for zoonotic spread of these microorganisms, besides the emergence of resistant and multidrug‐resistant pathogens among animal isolates (Chambers & DeLeo, 2009). Therefore, the present study evaluated the antimicrobial susceptibility profile and genetic diversity of *Staphylococcus* spp. isolated from dairy cattle with mastitis and observed a disturbing number of isolates resistant to penicillins, folate pathway inhibitors, and tetracyclines, further a great genetic heterogeneity in PFGE genotypes among the isolates.

**Figure 1:**
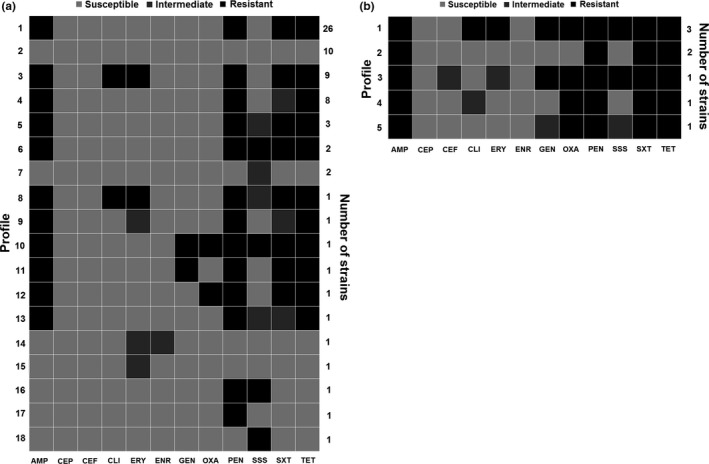
Antimicrobial susceptibility profile of *Staphylococcus aureus* (*n* = 71) (a) and coagulase‐negative staphylococci (CoNS) (b) isolated from milk samples of dairy cows with mastitis in Minas Gerais State, Brazil. Resistant (black), intermediate (dark gray), and susceptible (light gray). AMP: Ampicillin; CEP: cephalothin; CEF: ceftiofur; CLI: clindamycin; ERY: erythromycin; ENR: enrofloxacin; GEN: gentamycin; OXA: oxacillin; PEN: penicillin G; SSS: sulfonamide; SXT: trimethoprim/sulfamethoxazole; TET: tetracycline

The *S. aureus* and CoNS isolates exhibited full susceptibility only to cephalothin. The classification of *S. aureus* and CoNS isolates in susceptible, resistant, and intermediate to the twelve antimicrobials agents tested is shown in the Table 1. The susceptibility profile of *Staphylococcus* spp. isolated from dairy cows is shown in Table 2. Twelve [16.90% (12/71)] *S. aureus* were classified as multidrug resistance strains, whereas among CoNS isolates, the percentage of multidrug‐resistant isolates was 50.00% (4/8) (Figure 1). The percentage of oxacillin resistance was also higher among CoNS strains [75% (6/8)], compared to *S. aureus* isolates (MRSA) [2.81% (2/71)] (Figure 1). The susceptibility patterns of *S. aureus* ATCC 25923^T^ were in accordance with CLSI guidelines.

**Table 1:** Susceptibility of *Staphylococcus* spp. isolated from milk samples of dairy cows with mastitis from municipalities of the Zona da Mata Region, Minas Gerais, Brazil to 12 antimicrobial agents, 2010–2011

Antimicrobial	Zone diameter cutoff (mm)			*S. aureus*			CoNS		
	S	I	R	S (%)	I (%)	R (%)	S (%)	I (%)	R (%)
Ampicillin	≥29	–	≤28	17 (23.9)	NA	54 (76.1)	0 (0.0)	NA	8 (100.0)
Cephalothin	≥18	15–17	≤14	71 (100.0)	0 (0.00)	0 (0.00)	8 (100.0)	0 (0.0)	0 (0.0)
Ceftiofur	≥21	18–20	≤17	71 (100.0)	0 (0.00)	0 (0.00)	7 (87.5)	1 (12.5)	0 (0.0)
Clindamycin	≥21	15–20	≤14	61 (85.9)	0 (0.00)	10 (14.1)	4 (50.0)	1 (12.5)	3 (37.5)
Erythromycin	≥23	14–22	≤13	58 (81.7)	3 (4.2)	10 (14.1)	4 (50.0)	1 (12.5)	3 (37.5)
Enrofloxacin	≥23	17–22	≤16	70 (98.6)	1 (1.4)	0 (0.0)	8 (100.0)	0 (0.0)	0 (0.0)
Gentamicin	≥16	13–15	≤12	69 (97.2)	0 (0.0)	2 (2.8)	3 (37.5)	1 (12.5)	4 (50.0)
Oxacillin	≥18	–	≤17	69 (97.2)	NA	2 (2.8)	2 (25.0)	NA	6 (75.0)
Penicillin	≥29	–	≤28	15 (21.1)	NA	56 (78.9)	0 (0.0)	NA	8 (100.0)
Sulfonamide	≥17	13–16	≤12	59 (83.1)	7 (9.9)	5 (7.0)	3 (37.5)	1 (12.5)	4 (50.0)
Tetracycline	≥19	15–18	≤14	17 (23.9)	0 (0.00)	54 (76.1)	0 (0.0)	0 (0.0)	8 (100.0)
Trimethoprim/Sulfamethoxazole	≥16	11–15	≤10	17 (23.9)	10 (14.1)	44 (62.0)	0 (0.0)	0 (0.0)	8 (100.0)

**Table 2:** Antimicrobial susceptibility profiles found in *Staphylococcus* spp. strains isolated from milk samples of dairy cows with mastitis from municipalities of the Zona da Mata Region, Minas Gerais, Brazil, 2010–2011

Profile[Link]	Antimicrobials												*n* [Link]
	AMP	CEP	CEF	CLI	ERY	ENR	GEN	OXA	PEN	SSS	SXT	TET	
1	R[Link]	S[Link]	S	S	S	S	S	S	R	S	R	R	28
2	S	S	S	S	S	S	S	S	S	S	S	S	10
3	R	S	S	R	R	S	S	S	R	S	R	R	9
4	R	S	S	S	S	S	S	S	R	S	I	R	8
5	R	S	S	R	R	S	R	R	R	R	R	R	3
6	R	S	S	S	S	S	S	S	R	I[Link]	R	R	3
7	R	S	S	S	S	S	S	S	R	R	R	R	2
8	S	S	S	S	S	S	S	S	S	I	S	S	2
9	R	S	I	S	I	S	R	R	R	R	R	R	1
10	R	S	S	R	R	S	S	S	R	I	R	R	1
11	R	S	S	I	S	S	S	R	R	S	R	R	1
12	R	S	S	S	I	S	S	S	R	S	I	R	1
13	R	S	S	S	S	S	R	R	R	R	R	R	1
14	R	S	S	S	S	S	R	S	R	S	R	R	1
15	R	S	S	S	S	S	I	R	R	I	R	R	1
16	R	S	S	S	S	S	S	R	R	S	R	R	1
17	R	S	S	S	S	S	S	S	R	I	I	R	1
18	S	S	S	S	I	I	S	S	S	S	S	S	1
19	S	S	S	S	I	S	S	S	S	S	S	S	1
20	S	S	S	S	S	S	S	S	R	R	S	S	1
21	S	S	S	S	S	S	S	S	R	S	S	S	1
22	S	S	S	S	S	S	S	S	S	R	S	S	1

Notes

Susceptibility profiles highlighted in gray exhibited resistance to three or more classes of antimicrobials, being considered multidrug‐resistant profiles.

AMP: ampicillin; CEP: cephalothin; CEF: ceftiofur; CLI: clindamycin; ERY: erythromycin; ENR: enrofloxacin; GEN: gentamycin; OXA: oxacillin; PEN: penicillin G; SSS: sulfonamide; TET: tetracycline; SXT: trimethoprim/sulfamethoxazole.

Susceptibility profiles to twelve antimicrobials tested.

Number of isolates with identical susceptibility profile.

Resistant.

Susceptible.

Intermediate.

The high frequency of resistance to some of the main antimicrobial drugs such as penicillins, folate pathway inhibitors (sulfonamides), and tetracyclines (Table 1) can be explained by their frequent use in veterinary practice in recent years, especially among cattle. In fact, data from United States and from all European Union countries shown that tetracycline, penicillins, and sulfonamides are the three classes of antimicrobial most sold in those countries for veterinary use (EMA 2016; FDA 2015). Although there are no similar statistics for Brazil, it is likely that this scenario is the same in the country, since the practices with livestock animals are very comparable among these countries.

The percentage of resistance and multidrug resistance were markedly higher among CoNS isolates in comparison with *S. aureus* isolates (Tables 1 and 2). Among CoNS, a high rate of resistant (≥50%) to seven of the twelve antimicrobial tested was observed, which is of special concern, since this agent is ubiquitously distributed in nature among several species inhabiting different ecosystems and have become increasingly recognized as an important agent of nosocomial infection (Piette & Verschraegen, 2009). However, it is important to take into account that given the small sample size of CoNS isolates, it is difficult to draw clear conclusions about susceptibility profiles in this population. Anyway, albeit not surprising, the large amount of resistant, multidrug‐resistant, and oxacillin‐resistant isolates among CoNS deserves major attention, due to the potential risk of animal‐to‐human transmission of these strains. Indeed, it is important to consider that these microorganisms were found in milk and the majority of the Queijo Minas Artesanal, hand‐made cheese produced in the state of Minas Gerais, are made from raw milk, highlighting the potential public health hazards related to the consumption of CoNS‐contaminated food (Costa Sobrinho et al., 2012). Moreover, a recent study conducted in Minas Gerais showed a high count (10^6^–10^7^ CFU/g) and a high frequency of resistant and multidrug‐resistant CoNS isolated from soft cheese (Minas cheese) (Fontes et al., 2013), reinforcing that the food chain can be considered an important source for antibiotic‐resistant bacteria to human populations.

**Figure 2:**
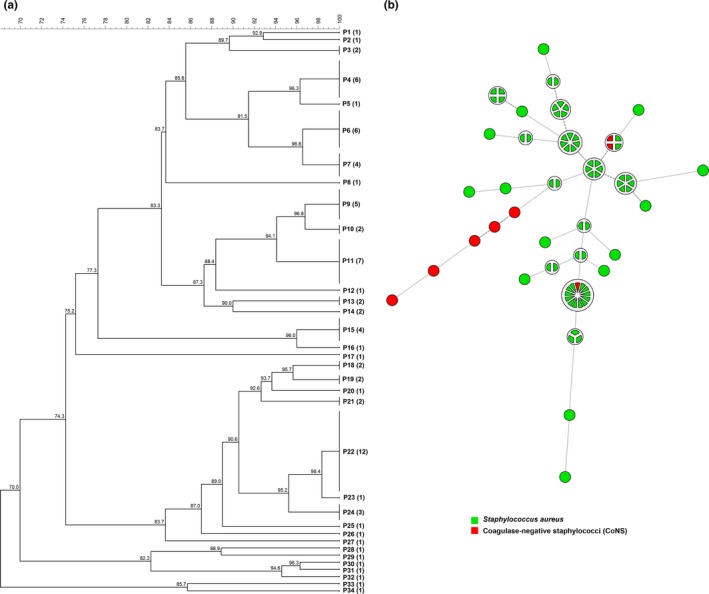
Dendrogram (a) and minimum‐spanning tree (MST) (b) of the 79 isolates of *Staphylococcus* spp. isolated from cows with mastitis in dairy herds in Minas Gerais state using PFGE data. The numbers in parentheses correspond to the number of isolates within each PFGE profile (P). The MST presented is the one with the highest overall reliability score and was calculated using UPGMA (unweighted pair group method using arithmetic averages) associated with the priority rule and the permutation resampling

Similarly to what was observed in other studies, in addition to oxacillin, all our MRSA isolates were also resistant to penicillin and tetracycline (Bardiau et al., 2013; Fessler et al., 2010). The low frequency of MRSA strains among *S. aureus* isolates from bovine mastitis observed in the present study has also been commonly reported elsewhere (Bardiau et al., 2013; Fessler et al., 2010; Moon et al., 2007). However, even at low frequency, it is worth noting that livestock‐associated MRSA (LA‐MRSA) can also contribute to human MRSA infections, which will vary in importance depending on other epidemiological variables present, as consumption of raw milk and milk products. In addition to resistance to all beta‐lactams, isolates resistant to three or more antimicrobial drugs were also considerably observed, representing 11 of the 22 different antimicrobial susceptibility profiles depicted (Figure 2). As the antimicrobial used in animal production is the same used to treat several zoonotic infections in humans, these finding strengthen the argument in favor of the prudent use of antibiotics by farmers and veterinarians.

Adopting 100% of similarity, 34 different genotypes were identified among the 79 *Staphylococcus* spp. isolates evaluated by PFGE. Figure 2 shows the cluster analysis (dendrogram and MST) of the PFGE fingerprint of the 79 *Staphylococcus* spp. isolates isolated from cows with mastitis in dairy herds from municipalities of the Zona da Mata Region, Minas Gerais. The HGDI calculated for the PFGE was 0.95. The association of MST analysis with data from municipality, herd, antimicrobial susceptibility pattern, and MRSA for all isolates did not show any clustering pattern (data not shown). However, a clustering pattern between bacterial species was observed; most of CoNS clustered together and distant from *S. aureus* isolates.

In this study, the PFGE was the method chosen to genotype *Staphylococcus* spp. isolates due its typeability and discriminatory power. The results observed on PFGE fingerprint of *Staphylococcus* spp. isolates from mastitis indicate a high genetic diversity among the isolates, despite the low number of herds tested (eleven). Although epidemiological information on animal traffic was not available, it is tempting to speculate that the great diversity of genotypes demonstrated could result from the intense traffic of animals among different herds, as it has been shown that the most of dairy herds in Minas Gerais state frequently sell or buy animals (Alves, 2009).

Furthermore, considering this high genetic diversity observed among the *S. aureus* isolates tested, it was not possible to establish a relationship between the genotype achieved and the origin of the isolates (herd and municipality), antimicrobial susceptibility profile, or MRSA. The large number of different genotypic profiles obtained among the isolates precludes the observation of any clustering pattern. Other possible explanation for the absence of relationships between the pulsetypes and herds, municipalities, or antimicrobial susceptibility profiles may have occurred due to the actual absence of association between these factors and genotypes, or could be the result of the low representative sampling considering all those categories. The last hypothesis seems more likely, since *S. aureus* is a contagious pathogen and thereby cattle movement would increase the probability of similar strains between neighboring farms or that have trade. However, regarding the bacterial species, it was possible to observe a clustering pattern as most of CoNS clustered together and distant from *S. aureus* isolates (Figure 1). These results support the usefulness of PFGE in molecular epidemiological studies of *Staphylococcus* spp., since it shows the correct judgment of related isolates without lose discriminatory power, which is a key characteristic of typing systems and is based on an estimate of their capability to differentiate between two unrelated isolates. Indeed, a high HGDI was observed among the typed *Staphylococcus* spp. isolates in the present study. Moreover, PFGE could assign a type to all *Staphylococcus* spp. isolates tested, showing its good typeability. These tools are essential for elucidating the pathogen's transmission chains and consequently to understand the distribution of the groups of microorganisms within and between herds, hosts, and reservoirs, as well as their phylogenetic relationships.

Overall, the results of the present study indicated a high frequency of antimicrobial resistance, especially among CoNS, and a high PFGE‐genetic diversity among *Staphylococcus* spp. strains isolated from dairy cows with mastitis in the Zona da Mata Region, Minas Gerais. Moreover, this study also provides insights on the pulsotypes of *Staphylococcus* spp. circulating in the State of Minas Gerais, which certainly contribute to a better understanding of the epidemiology and control of bovine mastitis in the state.

## CONFLICT OF INTEREST

The authors declare that they have no conflict of interest.

## AUTHORS CONTRIBUTION

ASG, MAVPB, and MBH conceived the study. EMSD, ASG, APL, and MBH participated in design of the study and wrote the paper. MDAMF, JAPA, CRP, and HBM participated in data acquisition and analysis. All authors read and approved the final manuscript.

## ETHICS STATEMENT

This research did not involve any human or animal subjects and therefore did not require any ethics oversight or approval in these respects.

## Data Availability

All data are provided in full in the results section of this paper.
